# Identifying Behaviors Predicting Early Morning Emotions by Observing Permanent Supportive Housing Residents: An Ecological Momentary Assessment

**DOI:** 10.2196/10186

**Published:** 2019-02-07

**Authors:** Rajesh R Nandy, Karabi Nandy, Emily T Hébert, Michael S Businelle, Scott T Walters

**Affiliations:** 1 Department of Biostatistics and Epidemiology School of Public Health University of North Texas Health Science Center Fort Worth, TX United States; 2 Oklahoma Tobacco Research Center The University of Oklahoma Health Sciences Center Oklahoma City, OK United States; 3 Department of Family and Preventive Medicine The University of Oklahoma Health Sciences Center Oklahoma City, OK United States; 4 Department of Health Behavior and Health Systems School of Public Health University of North Texas Health Science Center Fort Worth, TX United States

**Keywords:** permanent supportive housing, circumplex model of affect, ecological momentary assessment, emotion, valence, arousal, hierarchical mixed effects model, mobile phone

## Abstract

**Background:**

Behavior and emotions are closely intertwined. The relationship between behavior and emotions might be particularly important in populations of underserved people, such as people with physical or mental health issues. We used ecological momentary assessment (EMA) to examine the relationship between emotional state and other characteristics among people with a history of chronic homelessness who were participating in a health coaching program.

**Objective:**

The goal of this study was to identify relationships between daily emotional states (valence and arousal) shortly after waking and behavioral variables such as physical activity, diet, social interaction, medication compliance, and tobacco usage the prior day, controlling for demographic characteristics.

**Methods:**

Participants in m.chat, a technology-assisted health coaching program, were recruited from housing agencies in Fort Worth, Texas, United States. All participants had a history of chronic homelessness and reported at least one mental health condition. We asked a subset of participants to complete daily EMAs of emotions and other behaviors. From the circumplex model of affect, the EMA included 9 questions related to the current emotional state of the participant (happy, frustrated, sad, worried, restless, excited, calm, bored, and sluggish). The responses were used to calculate two composite scores for valence and arousal.

**Results:**

Nonwhites reported higher scores for both valence and arousal, but not at a statistically significant level after correcting for multiple testing. Among momentary predictors, greater time spent in one-on-one interactions, greater time spent in physical activities, a greater number of servings of fruits and vegetables, greater time spent interacting in a one-on-one setting as well as adherence to prescribed medication the previous day were generally associated with higher scores for both valence and arousal, and statistical significance was achieved in most cases. Number of cigarettes smoked the previous day was generally associated with lower scores on both valence and arousal, although statistical significance was achieved for valence only when correcting for multiple testing.

**Conclusions:**

This study provides an important glimpse into factors that predict morning emotions among people with mental health issues and a history of chronic homelessness. Behaviors considered to be positive (eg, physical activity and consumption of fruits and vegetables) generally enhanced positive affect and restrained negative affect the following morning. The opposite was true for behaviors such as smoking, which are considered to be negative.

## Introduction

More than half a million individuals are homeless at any given time in the United States [1]. Homelessness is associated with a higher prevalence of mental illness, higher rates of morbidity and mortality, and increased rates of drug abuse, criminality, and victimization [2]. Permanent supportive housing (PSH) is one approach to reducing chronic homelessness and provides low-cost community-based housing alongside supportive services. PSH has been demonstrated to reduce homelessness, increase housing tenure, and decrease emergency room visits and hospitalization [3]. Although PSH can result in lower overall costs to society, people who reside in PSH face numerous challenges in their ability to live independently, including, in many cases, physical and mental health conditions requiring treatment. For example, 73% of PSH residents in Fort Worth, Texas, United States, reported at least one chronic health condition, 55% reported having received treatment for a mental health condition, 67% reported having a history of substance abuse, and 44% reported both co-occurring substance abuse and mental health concerns [4].

Mood and emotional reactivity play an important role in both mental and physical health. For example, Gallo and Matthews [5] found that negative emotions and cognitions were related to cardiovascular disease and all-cause mortality and contributed to the relationship between socioeconomic status and health [6]. A study of well-being among adults in England associated positive affect with survival, even after controlling for demographic factors and baseline health [7]. In addition, multiple studies have shown the association of anxiety, stress, and negative affect with health behaviors such as smoking, alcohol, and drug use [8-12]. Additional research is needed to examine how health behaviors effect affect and stress in disadvantaged and understudied adults.

Ecological momentary assessment (EMA) techniques use mobile devices to assess thoughts, feelings, and behaviors in real-time in an individual’s natural setting [13]. A review of EMA studies on mood disorders and dysregulation demonstrated that real-time assessment reduces recall bias and allows for the study of dynamic processes and context-specific relationships related to mood [14]. For instance, in one 4-day long EMA study of depression among adolescents, higher pretreatment positive affect, lower negative affect, and a higher positive-to-negative affect ratio predicted a lower clinician-rated severity of problems following treatment [15]. The measures of affect were created using items adapted from the Positive and Negative Affect Scale for Children [16]. Similarly, another EMA study of affect and depressive illness found that response to treatment was predicted by daily increases in positive affect among individuals with clinical depression [17]. EMAs involving substance use in adolescents indicate that alcohol intake and cigarette intake are predicted by greater negative mood states, including sadness, depression, anger, and stress, as well as greater conduct and behavioral problems [18-21]. Overall, EMAs may be a useful way to monitor and, ultimately, intervene to prevent maladaptive mood experience and mood regulation processes [22].

Although EMA has been used to evaluate dynamic changes in mood and behavior, no study to date has examined the relationship between emotions and behavior among adults in PSH. The purpose of this study was to explore the prospective associations between emotions (ie, valence and arousal) and health behaviors among adults residing in PSH using EMA. Considering the relatively high costs associated with physical and mental health disorders in this population, it can be beneficial to identify factors affecting daily emotion patterns in order to predict and intervene with persons who are at risk.

## Methods

### Participants, Design, and Study Procedures

We obtained data for this study from the Mobile Community Health Assistance for Tenants (m.chat) project, a technology- assisted health coaching intervention designed to improve health indicators among PSH residents in Fort Worth, Texas [23]. We recruited participants via convenience sampling from 6 local housing agencies in Fort Worth. The participants were adult, English speaking, Medicaid-enrolled or eligible, and reported at least one of the following conditions in the past year: prescribed medication for psychological or emotional problems, experienced hallucinations, received a pension for a psychiatric disability, or reported at least moderate levels of depression (>9 on the Patient Health Questionnaire). Participants met monthly with a health coach who helped to set goals related to diet, exercise, substance use, medication compliance, social support, and recreation or leisure. We gave the opportunity to participate in the EMA portion of the project to a subgroup of participants who scored ≥4 on the Rapid Estimate of Adult Literacy in Medicine-Short Form (indicating >6th grade English literacy level). This subset of clients completed EMAs each morning with questions about current emotions and setting as well as health behaviors from the previous day, including diet, exercise, substance use, leisure time activities, medication compliance, and social interactions. We used an EMA protocol based on that of a previous study with homeless smokers [20]. We provided participants with a smartphone and granted unlimited voice, short message service text, and 2 GB data for their personal use. While enrolled in the EMA portion of m.chat, participants received up to 15 “Chat Bucks” each month, proportional to the percentage of days they completed the assessment (1 Chat Buck=US $1 redeemable for health-related supplies; thus, participants could earn up to US $15 worth of health-related supplies each month). Provided they were compliant with at least 50% of the EMA prompts, participants could carry the phone for up to 12 months. Project resources allowed for up to 80 participants to participate in the EMA portion at any one time; when participants returned the phone (because they had reached the end of their allotted time, were failing to complete assessments, or decided they did not want to carry the phone any longer), it was reset to factory settings and offered to another participant based on the order of enrollment into the parent study.

The Institutional Review Board of the University of North Texas Health Science Center approved this project, and we assured participants of confidentiality. All participants gave informed consent.

For analysis, we included 155 participants who completed a total of 18,357 daily assessments between May 1, 2016, and April 30, 2017. On average, individuals received 139 daily assessments or prompts (range 14-334) and completed 106 assessments (range 4-322). The sample was split almost evenly between males (n=77) and females (n=78), and the average age was 52 (SD 8) years.

### Instruments and Measures

The mobile app alerted participants to complete an assessment 30 minutes after the participant’s self-reported waking time. We asked the participants to complete the assessment within 30 minutes of the initial alert; they had the option to “snooze” an assessment request 3 times each day before the EMA would be counted as missed. Below are the questions that were presented in the daily EMA (we have only presented the questions or response options considered in the analyses).

#### Emotions

We measured 9 emotions items on a Likert-type scale from 1 (strongly disagree) to 5 (strongly agree): I feel happy, I feel frustrated, I feel sad, I feel worried, I feel restless, I feel excited, I feel calm, I feel bored, and I feel sluggish.

#### Physical Activity

Participants were asked how many hours they spent sitting, how many minutes they walked or biked to get somewhere, how many minutes they were physically active for fitness (eg, running or sports), and how many minutes they were physically active at work or home (eg, cleaning, lifting, or carrying things) the previous day.

#### Diet

We asked participants how many servings of fruits and vegetables they ate, how many sugar-sweetened beverages they drank, and how many desserts and other sweets they ate the previous day.

#### Social Support

We asked the participants about total minutes they spent in meaningful one-on-one conversations with other people and the total minutes they spent in meaningful group interactions (eg, going to church, participating in an exercise class, or other social occasions) the previous day.

#### Medication Compliance

We asked participants whether they took all of their medication as prescribed the previous day.

#### Tobacco Use

We asked participants whether they used tobacco (cigarettes) the previous day, and if so, how many cigarettes they smoked.

Demographic characteristics such as age, sex, and race (white or nonwhite), collected at baseline, were used as covariates in the analyses.

### Statistical Modeling and Analysis

**Figure 1:**
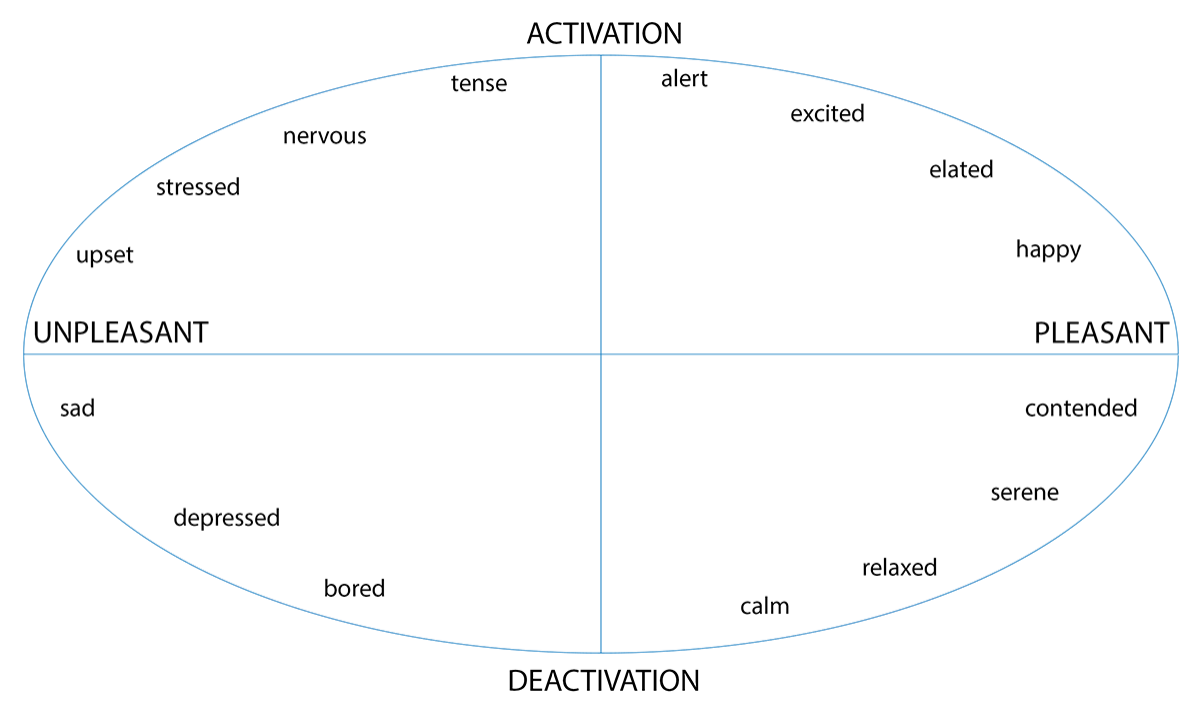
Circumplex model of affect.

**Figure 2:**
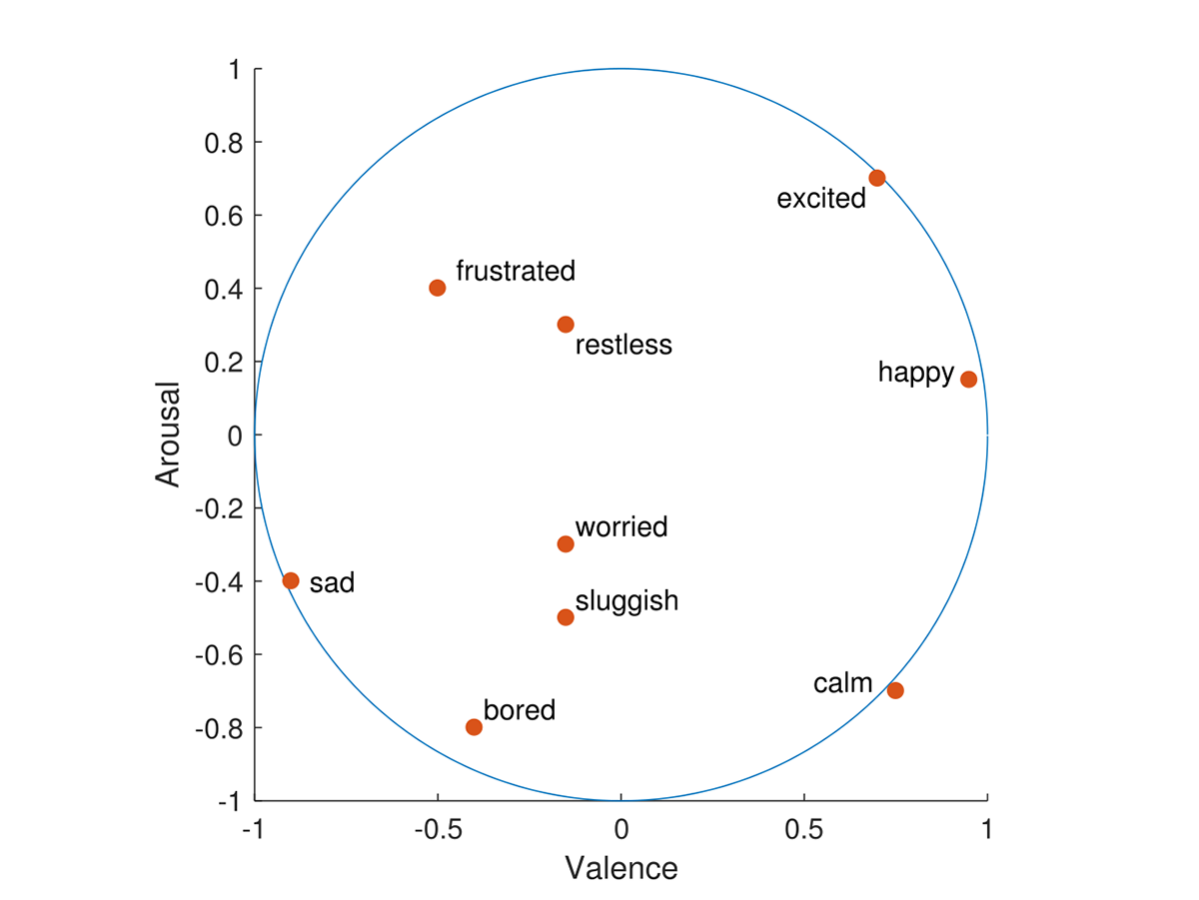
Circumplex model in Cartesian coordinate system.

The circumplex model of affect [24,25] was used to categorize each emotion in a 2-dimensional circular space, containing dimensions for arousal and valence (Figure 1). Valence, in the context of emotions, is defined as the intrinsic attractiveness or averseness of an event, object, or situation. Likewise, arousal is the state of being physiologically and mentally alert, awake, and attentive. In a recent refinement of the model using regression [26], the circumplex model was quantitatively visualized as a circular space of radius of 1 unit within a 2-dimensional Cartesian coordinate system, which assigns scores for valence and arousal for each emotion. This model includes a comprehensive list of mood items commonly considered in behavioral sciences. We obtained scores for valence and arousal in the circumplex from this model for each of the 9 emotion items considered (Table 1). In Figure 2, these 9 emotions are presented within the circumplex, depicting their valence and arousal coordinates. During each daily assessment, we created composite scores of valence and arousal as weighted sums of responses from all 9 emotion questionnaire items, with the reported emotion scores serving as weights. These two composite scores were the outcomes of this study. Specifically, let a subject’s response in Likert-type scale for I feel happy, I feel frustrated, I feel sad, I feel worried, I feel restless, I feel excited, I feel calm, I feel bored, and I feel sluggish be denoted by *l* 1, *l* 2, *l* 3, *l* 4, *l* 5, *l* 6, *l* 7, *l* 8, and *l* 9, respectively. Then, using Table 1, the composite score for valence is 0.95× *l* 1−0.5× *l* 2−0.95× *l* 3−0.15× *l* 4−0.15× *l* 5+0.7× *l* 6+0.75× *l* 7−0.4× *l* 8−0.15× *l* 9. The corresponding composite score for arousal is 0.15× *l* 1+0.4× *l* 2−0.4× *l* 3−0.3× *l* 4+0.3× *l* 5+0.7× *l* 6−-0.7× *l* 7−0.8× *l* 8−0.5× *l* 9. In Table 2, for each emotion, we have provided the mean of the subject means, SD of the subject means (between subjects), and mean of the subject SDs (within subjects). All questions in the various domains (eg, diet and physical activity) described in the Instruments and Measures section, except the emotions items, were considered as potential predictors of the outcomes. To reduce the large number of predictors in the model, we combined some of the variables within the same domain to create the following new variables as predictors: number of servings of healthy diet, number of servings of sweets, and number of minutes of total physical activity. In Table 3, for each momentary predictor, we have provided the mean of the subject means, SD of the subject means (between subjects), and mean of the subject SDs (within subjects). It is important to observe that even though the EMA emotions questions asked about present emotions, predictors were recalled values from the previous day.

**Table 1:** Circumplex scores for the emotions considered.

Emotion	Valence	Arousal
Happy	0.95	0.15
Frustrated	−0.50	0.40
Sad	−0.95	−0.40
Worried	−0.15	−0.30
Restless	−0.15	0.30
Excited	0.70	0.70
Calm	0.75	−0.70
Bored	−0.40	−0.80
Sluggish	−0.15	−0.50

**Table 2:** Descriptive statistics for the 9 emotion outcomes.

Variable	Mean	Between-subject SD	Mean within-subject SD
Happy	3.54	0.74	0.70
Sad	2.55	0.79	0.79
Restless	2.59	0.83	0.70
Excited	3.09	0.78	0.70
Calm	3.42	0.71	0.67
Sluggish	2.71	0.92	0.74
Frustrated	2.55	0.79	0.79
Worried	2.60	0.85	0.74
Bored	2.41	0.80	0.66

**Table 3:** Descriptive statistics for the quantitative momentary predictors.

Variable	Mean	Between-subject SD	Mean within-subject SD
Total physical activity	34.62	24.67	20.78
Minutes of one-on-one interaction	79.97	49.28	48.94
Minutes spent in group interaction	44.41	32.74	36.97
Hours spent sitting	5.47	1.93	2.13
Fruits and vegetables	2.80	1.55	1.23
Sweets	2.60	1.67	1.13
Number of cigarettes	3.49	5.12	1.66

In that sense, the predictors are not strictly momentary, but will be referred to as momentary variables for statistical modeling and analysis. Individual demographic characteristics (ie, age at the onset of the EMA study, sex, and race) were considered time invariant for the duration of the study. Race was dichotomized as white and nonwhite, as 94.5% (147/155) of participants were either white or African American individuals.

In the general statistical model for the analysis, for each outcome, we denote the response on the *t*^*th*^ assessment from the *i*^*th*^ subject by *Y*_*it*_, the value of the *j*^*th*^ demographic predictor (out of *k* total number of predictors) from the *i*^*th*^ subject by *X*_*ij*_ and the value of the momentary predictor on the *t*^*th*^ assessment from the *i*^*th*^ subject by *Z*_*it*_. Then the hierarchical model can be presented as follows:

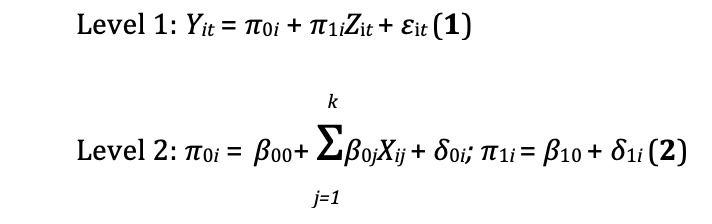



All analyses were performed using MIXED procedure in SAS (SAS Institute) with the intercept specified as a random effect and within-subject residuals specified to have a first-order autoregressive correlation.

Since there are eleven predictors in our model, we implemented the popular Bonferroni correction to adjust the reported *P* values for the predictors. It should be noted that the very conservative Bonferroni-corrected *P* value threshold of .05 is equivalent to an unadjusted *P* value threshold of .0045. Since we were not interested in the statistical significance of the intercept term, we did not consider it for the Bonferroni correction.

## Results

### Associations Among Momentary Variables and Emotions Controlling for Demographic Characteristics

Analyses of the associations between momentary variables and valence and arousal were performed, controlling for the 3 demographic predictors of age, sex, and race. The results for the valance and arousal outcomes are presented in Tables 4 and 5, respectively (all the *P* values correspond to 2-tailed tests). For the predictors with an unadjusted *P* value<.05, we also presented Bonferroni-corrected *P* values (in parentheses). None of the demographic variables predicted either outcome at a statistically significant level after the extreme Bonferroni adjustment. However, the effect of race on valence barely missed significance after adjustment (unadjusted *P* value=.007; adjusted *P* value=.08), with white individuals reporting much lower valence scores on average.

Minutes spent doing physical activity the previous day was a statistically significant predictor of both valence and arousal, with expected higher scores for increased physical activity. Time spent in meaningful group interaction the previous day was not a statistically significant predictor of either valence or arousal. Time spent in meaningful one-on-one social interaction the previous day was a statistically significant predictor of both valence and arousal, with expected higher scores for more interaction time. Hours spent sitting the previous day was a statistically significant predictor of both valence and arousal, with an expected lower score for an increase in time spent sitting. Number of total servings of fruits and vegetables consumed the previous day was a statistically significant predictor of both valence and arousal, with expected higher scores for greater servings. Number of total servings of sugar-sweetened beverages and desserts the previous day was not a statistically significant predictor of either valence or arousal. Adherence to medication the previous day was a statistically significant predictor of both valence and arousal, with higher scores for adherence. Any tobacco usage the previous day was a statistically significant predictor of only valence; on average, smoking a higher number of cigarettes resulted in lower valence scores.

**Table 4:** Results for valence with momentary predictors, controlling for demographic characteristics.

Effect	Estimate	SE	*t (df)^a^*	*P* value^b^
Intercept	2.32	1.422	1.63 *(152)*	.10
Age	−0.008	0.027	−0.28 *(153)*	.78
Male	−0.49	0.459	−1.07 *(153)*	.28
Caucasian	−1.23	0.457	−2.69 *(153)*	.007 (.08)
Total physical activity	0.007	0.0008	8.80 *(153)*	<.001 (<.001)
Minutes of one-on-one interaction	0.004	0.0004	9.53 *(153)*	<.001 (<.001)
Minutes spent in group interaction	0.00008	0.0005	0.17 *(153)*	.87
Hours spent sitting	−0.04	0.011	−3.42 *(153)*	<.001 (.007)
Fruits and vegetables	0.12	0.014	8.54 *(153)*	<.001 (<.001)
Sweets	0.02	0.016	1.34 *(153)*	.18
Medication	0.76	0.105	7.23 *(153)*	<.001 (<.001)
Number of cigarettes	−0.06	0.008	−7.99 *(153)*	<.001 (<.001)

^a^All the *P* values correspond to 2-tailed tests.

^b^Bonferroni-corrected *P* values are in parentheses.

**Table 5:** Results for arousal with momentary predictors and controlling for demographic characteristics.

Effect	Estimate	SE	*t (df)^a^*	*P* value^b^
Intercept	−2.97	0.520	−5.72 *(152)*	<.001
Age	−0.0001	0.010	−0.01 *(153)*	.99
Male	−0.17	0.167	−1.04 *(153)*	.30
Caucasian	−0.42	0.167	−2.50 *(153)*	.01 (.13)
Total physical activity	0.003	0.0004	7.40 *(153)*	<.0001 (<.001)
Minutes of one-on-one interaction	0.002	0.0002	11.17 *(153)*	<.0001 (<.001)
Minutes spent in group interaction	0.0003	0.0002	1.67 *(153)*	.09
Hours spent sitting	−0.02	0.005	−4.20 *(153)*	<.001 (<.001)
Fruits and vegetables	0.03	0.006	4.07 *(153)*	<.001 (<.001)
Sweets	−0.009	0.007	−1.36 *(153)*	.17
Medication	0.15	0.045	3.26 *(153)*	.001 (.01)
Number of cigarettes	−0.006	0.003	−1.88 *(153)*	.06

^a^All the *P* values correspond to 2-tailed tests.

^b^Bonferroni-corrected *P* values are in parentheses.

### Influence of Demographic Variables on Momentary Predictors for Emotions

Even though the model controls for demographic characteristics in analyzing the effect of momentary variables on valence and arousal, it is worthwhile to explore how much influence the demographic predictors have on the momentary predictors. A strong influence of demographic predictors on the momentary predictors can make the regression coefficients unstable and hard to interpret. Unlike in a standard multiple regression framework, in our hierarchical model, the influence cannot be measured directly by studying the multicollinearity properties and other standard regression diagnostics. Instead, the amount of influence can be indirectly measured by analyzing two additional models: one with only demographic predictors and one with only momentary predictors. The change in values of the estimated regression coefficients in the full models compared with the two isolated models described above can be used to assess the influence and the robustness of the coefficients.

For the sake of brevity, we did not present the actual results from the two isolated models here, but the results are remarkably consistent with our findings from the combined model in the previous section. Not only do the statistical significances of the momentary predictors match closely but also the individual estimates of the regression coefficients are surprisingly close. The individual estimates of the regression coefficients are very close for the demographic predictors as well. The observed consistency provides fairly strong evidence on the orthogonality of the demographic predictors from the momentary predictors.

## Discussion

### Principal Findings

These findings provide an important glimpse into factors that affect valence and arousal in a population of individuals residing in supportive housing. To our knowledge, this is the first study to examine the connection between emotions and other factors among people with mental health disorders and a history of chronic homelessness. This underserved population is often excluded from research studies due to co-occurring mental and physical disorders, resulting in substantial gaps in our understanding of their health and health behaviors.

Our analyses provide a number of observations about the relationships between health behaviors and subsequent emotions. First, we found that physical activity was significantly associated with positive emotions the following day. This finding is consistent with the literature showing the association of moderate physical activity with improved and maintained mood [27] as well as decreased symptoms of depression and anxiety [28]. We also found a positive relationship between fruit and vegetable intake and emotions the following day. Similarly, a study using data from the Canadian Community Health Survey found a significant association of fruit and vegetable intake with lower odds of depression and psychological distress [29]. Taken together, these results argue that a coordinated program to improve physical activity and diet should be a fundamental part of health interventions for people with mental health disorders and a history of chronic homelessness.

We also found that smoking cigarettes had a negative effect on valence the following day. Although nicotine may have a calming effect due to the inhibition of negative emotions such as anger [30], our results suggest that this effect may not carry forward to the following day. Research has suggested that nicotine dependency exacerbates stress [31], and a meta-analysis of changes in mental health after smoking cessation revealed that smoking cessation is associated with reduced depression, anxiety, and stress, with effect sizes equal to or larger than those of antidepressant treatment for mood disorders [32]. Thus, individuals receiving treatment for mood disorders may benefit from concurrent smoking cessation therapy.

We also found a strong relationship between the amount of time spent in individual social interactions and emotions. Interestingly, “time spent on meaningful one-on-one social interaction the previous day” was strongly associated with arousal and valence, while the “amount of time spent interacting in a group setting” was not significantly associated with emotions. This finding was unexpected given the substantial evidence that social support predicts the quality of life in many areas [33]. However, supportive housing residents are encouraged to attend support groups that address lifestyle skills, chronic disease management, and substance use. For this population, it may be that group interactions do not contribute to emotions unless the individual feels personally connected to at least one other person in the group. Thus, group interactions by themselves may not predict emotions, while individual interactions outside of the group setting may be one indicator of healthy, rewarding relationships.

In the analysis of the effect of demographics on the association between momentary predictors with valence or arousal, demographic variables had minimal effects on the regression coefficients of the momentary predictors, even when statistically significant. Hence, it is reasonable to conclude that the demographic predictors operated almost independently of the momentary variables in terms of influencing emotions.

Finally, it would be possible to study the association of emotional affect with subsequent same-day behaviors, for instance, examining the effects of emotions in the morning on smoking or drinking later in the day. Such analyses are beyond the scope of this paper, but we plan to investigate these associations in a future manuscript.

### Limitations and Strengths

Our study had a number of limitations. Notably, our protocol included only daily morning assessments. Thus, we were not able to examine within-day variability. However, unlike other EMA studies, which typically run for a few days to, at most, a few weeks, our study ran up to 334 days with an average of 156 days of monitoring among all participants. This allowed us to examine associations for a much longer period than most other EMA studies. In addition, our results are generalizable only to a population of individuals residing in PSH with a history of homelessness and mental health issues. It is unclear whether the findings are generalizable to other groups of people with mental health problems, let alone the population in general. Relatedly, all participants self-reported depression or a mental health condition at baseline, which may have affected emotions and mood independently of other behavioral measures. For instance, the average client reported a score of 12.62 on the Patient Health Questionnaire, indicating that most clients felt at least moderate levels of depression upon admission to the program. Finally, we cannot rule out the possibility that participating in the coaching intervention affected the relationship between behaviors and emotion. Our results must be interpreted in the context of the larger services that people were receiving in this program. Further study with a more diverse population is necessary to make any broader assertions.

### Conclusions

Despite the limitations, our study offers an important glimpse into health behaviors that affect daily emotional arousal and valence of persons with a history of chronic homelessness and mental health problems. One of the goals of the m.chat program was to provide individual support and assistance in meeting health goals. Because mood was an important target of the program, identifying factors that predicted positive affect can help improve future iterations of programs like this. To that end, identifying modifiable behaviors associated with negative and positive moods is a first step toward improving stability and preventing future homelessness. Understanding factors associated with mood and behaviors, particularly in vulnerable populations such as formerly homeless individuals, can also help providers design more targeted treatment plans and provide more appropriate referrals to ancillary care services [34].

Notably, many of our findings are consistent with “common wisdom” drawn from other populations. Behaviors generally considered to be positive (eg, physical activities, consumption of fruits and vegetables, adherence to prescribed medication, and one-on-one social interaction) tended to enhance positive affect and restrain negative affect. The opposite was true for behaviors considered to be negative, such as smoking. In fact, the positive and negative impacts on physical health for most of these behaviors are well established, and it is noteworthy that their effects on positive and negative affect appear to be consistent with previous literature with other populations. In a separate analysis of m.chat program data, Holmes et al [35] found that participants who consumed the least Western-style foods (eg, fast food, sugar-sweetened beverages, and processed meat) had significantly lower depressive symptoms over 1 year than those who consumed the most Western-style foods. However, Holmes et al also reported no significant association between depressive symptoms and nutritious food intake and physical activity over 18 months. Because we found a relationship between fruits and vegetables, exercise, and next day mood (but not sugar and mood), the relationships between these variables may be somewhat different in the short versus long term. Such behaviors can be targeted with a goal of enhancing positive affect and restraining negative affect in order to improve the overall mental health of individuals. Such an intervention has the potential to improve treatments for individuals with mood and other psychological disorders.

## Abbreviations

EMA: ecological momentary assessment
PSH: permanent supportive housing
